# Longitudinal Effects of the Family Support Program Chancenreich on Parental Involvement and the Language Skills of Preschool Children

**DOI:** 10.3389/fpsyg.2020.01282

**Published:** 2020-06-12

**Authors:** Franziska Cohen, Juliane Schünke, Eric Vogel, Yvonne Anders

**Affiliations:** ^1^Lehrstuhl für Elementar- und Familienpädagogik, Otto-Friedrich-Universität Bamberg, Bamberg, Germany; ^2^Arbeitsbereich Frühkindliche Bildung und Erziehung, Freie Universität Berlin, Berlin, Germany

**Keywords:** family support program, home learning environment, vocabulary skills, grammar understanding, longitudinal

## Abstract

When they enter primary school children already vary significantly in their language skills, depending at least in part on their family’s social background. In particular, the home learning environment plays a significant role in children’s development. For that reason, early intervention programs have been developed to obviate learning difficulties and to promote health, children’s development, and educational equality. The family support program Chancenreich aims to encourage the interaction and relationship between parents and children through two different course formats. The present study examines the longitudinal effects of attending the Chancenreich program and different course formats on (a) parents attending further educational services for children after completing the program, (b) children’s vocabulary and level of grammar development at the age of 5 and (c) the children’s vocabulary development between the ages of 3 and 5. Furthermore, we examine the relationship between family characteristics and the attendance rates of different course formats of the Chancenreich program at the first and second point of measurement. The study follows a longitudinal design with two points of measurements (T1: M_age_ = 41 months, T2: M_age_ = 68 months), and a sample size of 121 parents and their children at T2 in the intervention group and 41 parents and their children in the comparison group. Findings indicate that attendance of the Chancenreich program’s courses is related to child and family characteristics and to later patterns of course participation after completing the program. Further, both children’s level of vocabulary skills (PPVT) at the age of 5 and their development between the ages of 3 and 5 benefit from the parental participation in parenting skills training at the age of 3. Implications and future research on the effectiveness of family support programs are discussed.

## Introduction

Language development is an important milestone for young children, and is relevant for their cognitive and socio-emotional competencies in early childhood and for later school success (Hoff, 2013). In particular, children’s vocabulary and their understanding of grammar are relevant for language use in daily conversation and emergent literacy (Ouellette, 2006; Swanson et al., 2008). However, children consistently show early differences in language skills that can be explained by the cultural and social background of their families (Hart and Risley, 1995; Ginsborg, 2006; Senechal, 2011; Hirsh-Pasek et al., 2015). In particular, the quality of the home learning environment (HLE) plays a major role in early development and later academic success (Melhuish et al., 2008; Rodriguez and Tamis-LeMonda, 2011; Skwarchuk et al., 2014; Tamis-LeMonda et al., 2017). Within the theoretical model of the HLE, researchers distinguish between structural characteristics, beliefs, and process quality, with process quality having a direct effect on children’s learning outcomes (Kluczniok et al., 2013). These findings have resulted in initiatives to increase the quality of families’ HLEs, thus creating early positive learning trajectories in order to prevent socially determined disparities in educational careers.

For that reason, early intervention programs have been developed to obviate learning difficulties and to promote health, children’s development, and educational equality (Campbell et al., 2002; Cadima et al., 2017; Heckman et al., 2013). Furthermore, these programs aim to promote parents’ knowledge, skills, and confidence and provide guidance on their children’s development, the parent–child relationship, and parenting practices. In conclusion, it can be assumed that supporting parents in providing a rich HLE for their young children will have beneficial effects on children’s early and later skill development. Furthermore, early positive experiences with family support services motivate parents to cooperate with and use further educational services in their children’s later life.

In Germany, empirical evidence on the effectiveness of family and child support programs is rare. Furthermore, existing evaluation studies have been limited to cross-sectional study designs, which do not enable the identification of causal relationships between the program and outcomes (Van der Stede, 2014). For this reason, we will investigate in this study the longitudinal effects of the family support program Chancenreich. This program offers services for families with young children up to the age of three. We examine first the effects of the program on families’ attendance rates at different course types and further educational services. Our second aim is to examine the effects of the program on the language development of children at the age of 5, up to 2 years after completing the program.

The paper begins by introducing the theoretical framework of the HLE that this study applies in order to understand the relationships between the different aspects of children’s environments and their effects on children’s language skills. Section two provides a brief research review of the characteristics of successful family support programs and their longitudinal effects on children’s language development. This chapter is followed by a description of the family support program Chancenreich and the different course formats. Finally, our research questions are presented. Consequently, we describe the design of our study and the methodological approach used, before presenting our results and discussing them with regard to the status of present research, its limitations, and implications for research, practice, and policy.

### Home Learning Environment

The underlying theory of the family’s HLE is defined by the developmental and living conditions in which a child is brought up, including the levels of familial support and encouragement of the child’s development (Lehrl, 2018). While many studies have explored and discussed the impact of the family’s HLE on children’s development (e.g., Gottfried et al., 1998; Melhuish et al., 2008; Niklas et al., 2015), few scientists have provided sound and comprehensive theoretical frameworks. According to Bronfenbrenner and Morris (2006) Process-Person-Context-Time (PPCT) model, children’s development is affected by contextual, personal, and proximal processes. More specifically, Kluczniok et al. (2013) provide a synthesis of different theoretical assumptions and describe the quality of the home environment as a multidimensional concept comprising three different dimensions: structural quality, parental beliefs, and process quality. The structural aspects of the HLE relate to stable, long-lasting characteristics pertaining to family background and composition (e.g., parental educational level, socio-economic and immigration status, and the availability of learning materials). The second dimension describes educational beliefs, for example the educational aspirations and values regarding a child’s upbringing and development. The third dimension – process quality – refers to activities and interactions between parents and their children, interactions among children, and the use of the spatial and material environment in the home. It is assumed that structural aspects and beliefs are directly related to process quality, which in turn directly affects the outcomes of children’s development. Furthermore, several studies have shown that structural disadvantages also correlate with fewer positive interactions and fewer enriching activities (Bradley et al., 2001; Sylva et al., 2004). However, numerous researchers argue that structural aspects of the home environment do not entirely predetermine process quality (Sylva et al., 2004; Bornstein and Bradley, 2008).

The concept of the family intervention program Chancenreich can be linked to the structural-process model of the HLE. While considering the background characteristics of the families, Chancenreich focuses on supporting families’ process quality as an important predictor for children’s developmental outcomes.

### Family Support Programs

Family support programs often comprise various approaches, e.g., house visits and parenting courses, that aim to promote parenting competences or support the parent–child relationship. First, these programs can be distinguished by their universal or target group approaches. Universal preventive programs are offered to all children and families without identifying the individual risk. In comparison, selective and indicated preventive interventions target families and children whose risk of developing difficulties or diseases is higher than average or who already face developmental problems (Mrazek and Haggerty, 1994).

Furthermore, Layzer et al. (2001) identify in their meta-analysis stronger positive effects for intervention programs which start earlier in children’s lives, before problematic behavior occurs, and which involve parents in training courses provided by professional staff. For example, the results of a meta-analytic review of parent training programs designed to enhance behavior and adjustment in children aged 0–7 showed positive stronger effects from courses that combine a direct targeting of parenting skills and a focus on positive parent–child interaction and communication skills (Kaminski et al., 2008). In addition, programs with longer duration, and a more frequent and regular attendance of intervention programs, seem to be a predictor for better child and parent outcomes (Ramey and Ramey, 1998; Halpern, 2000; Denham and Burton, 2003; Nievar et al., 2010). Intervention programs with a broad approach, offering different services to the parents and children, show greater effects on children’s outcomes than interventions that have a very narrow, focused goal. However, a broad approach might only be beneficial in the context of the methods and services offered, and not necessarily with regard to the targeted competences of the parents and children. Compared to these findings, Blok et al. (2005) establish no differentiating effects for program length, intensity, or long-term continuation.

Programs can be further distinguished by their delivery mode. Home-visit programs offer families tailored support in the context of their own homes, while center-based programs work directly with children in an institutionalized context. Research shows that the inclusion of home visits in the program may benefit children’s development and improve the HLE (Kendrick et al., 2000), even though the meta-analyses of Sweet and Appelbaum (2004) and Filene et al. (2013) show that no specific home-visit program characteristic was related to the variation of the effects. Blok et al. (2005) reveal in their meta-analysis that, in particular, the combination of center-based and home-based programs is an important success factor.

### Longitudinal Effects of Family Support Programs

One particular finding of longitudinal studies has been to establish the impressive cost-benefit advantages of early intervention programs (Shonkoff and Phillips, 2000; Karoly et al., 2005; Heckman, 2006) and positive detectable effects into adulthood (Reynolds et al., 2001; Nelson et al., 2003). However, we have little in-depth knowledge of these mechanisms and why temporary programs are still beneficial in later childhood.

On the one hand, Slavin et al. (1994) emphasize the beneficial effects of continuously interlinked support programs for children and families across age groups, although they note the difficulties in implementing them, since the early childhood education system is legally and organizationally fragmented due to different procedures in administration and funding (Reynolds et al., 2010). Ramey and Ramey (1998), on the other hand, hypothesize that skills developed earlier form the basis for future skills and, moreover, this skill base enables children to access and implement richer learning environments in a more efficient way. Furthermore, it is assumed that successful learning in early childhood may support the development of positive motivation and self-efficacy in children, promoting learning in later childhood as well. Similar effects can be assumed for the parents. Positive experiences in educational settings with very young children may encourage parents to become more involved in their children’s later educational careers by using and demanding more services (Epstein and Sanders, 2000). Parental involvement in their children’s development and education can also be transferred across settings (complementary learning). It can be assumed that parents who are familiar with the educational system are able to reduce uncertainty and to make good choices in the prospective educational careers of their children, particularly disadvantaged families who are usually underrepresented in involvement activities (Lösel, 2006; Dearing et al., 2009). Finally, parents who are interested and attentive with regard to their children’s education act as role models for their children.

To summarize, we assume that supporting parents in providing a rich HLE for their children throughout childhood, as well as cumulative participation in family support services, have beneficial effects on children’s skill development and parents’ attendance rates in other educational services.

A variety of professional interventions have been developed worldwide to support parents, promote parenting skills, and raise parental self-efficacy with regard to educational tasks (Cadima et al., 2017). Chancenreich is one example of a family support program in Germany and will be described in the following chapter.

### The Chancenreich Program

Chancenreich is a regional program implemented in Herford, a town in Germany (North Rhine-Westphalia). It aims to enhance parenting skills and child outcomes by offering a variety of services to all parents of children of up to 3 years of age (e.g., home visits, parenting courses), and is therefore considered a part of a universal approach. Chancenreich is unique in Germany for several reasons: (a) it is offered for free to all parents of newborns in the town, regardless of their social or cultural background; (b) it uses a modular approach consisting of many services with different content, from which parents can choose modules according to their needs; (c) Chancenreich offers a monetary incentive of €500 to all families who participate in at least five of these modules (Wilke et al., 2014). The five mandatory modules in the Chancenreich program relevant for the allocation of the monetary incentive are: use of home visits by pedagogical or pediatric staff, regular pediatric check-ups for the child, participation in a scientific evaluation of the program, enrolment of the child in an ECEC setting by the age of 3^1^, and the completion of the parenting training module. In this study, we focus on the specific effects of this parenting training module.

The parenting training module offers parents a variety of courses, which differ in their approach, content, length, and intensity of family participation. We categorized the different course formats according to their respective goals, content, and delivery format and labeled them ‘parenting-skills focused’ and ‘parent-child-interaction focused’ (Layzer et al., 2001).

### Parenting-Skills Focused Courses

Parenting-skills focused courses are attended exclusively by the parents. Courses of this type primarily focus on the parents and their parenting behaviors and skills. Hence, these courses usually have a stronger educational focus and parents receive feedback about their own behavior from other parents and from the instructor. Additionally, they are given input on their child’s development. Parents usually attend these courses for a set period, such as 8 or 10 weeks, and they visit the classes without their child. These courses are developed for parents with toddlers; they are based on cognitive behavioral theories or individual-psychological theories, and aim to help parents prevent child behavior problems (Nowak and Heinrichs, 2008). Examples of parenting-skills focused courses that are common in the Chancenreich program are Triple P and Strong parents – strong kids. Triple P is a widely-evaluated parenting course format which has been established to produce positive effects by reducing negative and inconsistent parenting practices (Sanders, 1999; Sanders et al., 2000, 2003). The parents’ course Strong Parents – Strong Children aims at strengthening parental skills and thus promotes the development and implementation of a non-violent upbringing. The results of an evaluation study showed that parents were able to increase their self-efficacy and positive parental behavior, reduce negative behavior patterns and parents rated the social behavior of their children as better after attending the course (Rauer, 2009). Previous research on parenting-skills focused courses has demonstrated that parents showed less dysfunctional parenting behavior and an increase in positive parenting behavior and parenting competency (de Graaf et al., 2008; Hahlweg et al., 2010). Early participation in these courses might have an early preventive effect, before a child begins to exhibit problematic behavior.

### Parent-Child-Interaction Focused Courses

Parent-child-interaction courses focus on the overall development of the child and include components that promote parent–child interaction and bonding, while also helping to build social networks among parents (Layzer et al., 2001; Thomas, 2013). Activities involving the participation of both the child and their parents^2^ are central components of these courses. These child-focused programs are usually offered to parents of infants and toddlers. This type of course is very popular in Germany: a third of parents with children below the age of 3 attends parent–child groups of this kind (Mühler and Spieß, 2008). The following courses are very well-received by parents and are therefore offered within the Chancenreich program:: Pekip (Höltershinken, 2011), Fun Baby (Tschöpe-Scheffler, 2006), or baby massage courses (Brisch and Hellbrügge, 2010).

Pekip (Prague Parent Child Program) and Fun Baby courses are aimed at parents with babies and toddlers. Together with other parents and babies, the motor skills, the baby’s senses and the parent–child interaction are developed in a playful way in a group. Participating parents of the Pekip courses reported, for example, that they are becoming increasingly sensitive to their children (Höltershinken, 2011). The baby massage courses, on the other hand, are designed to promote the baby’s healthy physical, social, and emotional development and foster a positive mother–child bond.

At the second point of measurement, the approach of these courses is adapted to the age of the children. Beyond the age of 3, parent-child-interaction focused courses typically consists of courses designed for joint activities between parents and their children, such as parent–child gymnastics.

Both the parent-child-interaction focused courses and parenting-skills focused courses are implemented in the parenting training module of Chancenreich, but it should be mentioned that they are part of the open educational market. In this case, such courses must be organized and financed by the parents themselves. This can be a challenge for many parents dealing with financial or social challenges, resulting in lower participation rates in such course formats. In comparison, the Chancenreich program offers these courses to all parents free of charge, organizes the courses close to the parents’ home and, for example, also reminds them of the next course session. As a result, his offers a lower threshold for disadvantaged parents to participate in such courses.

### Early-Education Focused Courses

Another course format becomes more relevant to a child’s life as they get older: early-education focused courses. These courses are not provided by the Chancenreich program, because the target age group of this format is beyond their age of interest (children from ages 3 and older). However, these courses become more relevant in preschool age, and need to be considered when investigating the potential effects of different courses both on the later usage of educational services and on children’s development.

Early-education focused courses consist of all types of adult-supervised activities for children that provide opportunities to develop specific skills or knowledge and take place outside the home or preschool. These courses are widely used and available on the educational market. There exists a broad range of activities that are included in this format, e.g., sport classes, early music education classes, creativity classes, and so on. In contrast to the parent-child-interaction focused courses, the child’s activity is central, with parents participating, but in a less active, more observational role. These courses are mostly offered to children from ages 3 and older, and research shows benefits of early-education focused courses for children’s socio-emotional and cognitive outcomes (Metsäpelto and Pulkkinen, 2014; Carolan, 2018).

### Research Questions

To understand the role of family background characteristics in predicting the selection of certain course types, we must first examine the following questions:

• What is the relationship between family characteristics and attendance rates of different course formats of the Chancenreich program at the first and second point of measurement?
  Assuming that parental participation in family support services in their children’s early years has beneficial effects on parental involvement in their child’s development and educational career (Slavin et al., 1994; Ramey and Ramey, 1998; Epstein and Sanders, 2000), we ask:
• What is the relationship between attendance rates for the Chancenreich program and further attendance of courses after completion of the program?
  While parent-child-interaction courses focus on the relationship between parents and children, with the children themselves participating in an activity, the participation in parenting-skill focused courses give parents the opportunity to reflect on their parenting behavior and to learn new successful strategies to improve process quality. For that reason, both of the program’s course types can be seen as a strategy for improving families’ process quality. Drawing on the theoretical model of the HLE, and research evidence on the effects of family support programs on children’s development, this paper’s other two key research questions are:
• What are the effects of the different course formats of the Chancenreich program on children’s levels of language development at the age of 5?
• What are the effects of the different course formats of the Chancenreich program on children’s language development between the ages of 3 and 5?

## Materials and Methods

### Design

The data come from the AQuaFam study, which examined the Chancenreich family support program. AQuaFam follows a longitudinal, quasi-experimental design with two points of measurement; it has an intervention group (families who attended the Chancenreich program) and a comparison group. The study consists of data from parents and their children. The data collection for the first time of measurement (T1) took place between November 2013 and May 2014, and for the second measurement (T2) between March and June 2016. For both times of measurement, trained research staff collected data from the families through a standardized family interview, a parents’ questionnaire, and by conducting standardized tests of each child’s language development at the family’s home. Parents have signed an informed consent form to participate in the study.

### Participants

The families of the intervention group were recruited in the town of Herford, Germany, where the Chancenreich program is based. Due to ethical considerations and for reasons of recruitment, families of the comparison group were recruited in a neighboring town through notices in early childcare settings, family education centers, pediatricians’ offices, and newsletters^3^.

At the first time of measurement (T1) in 2014, the sample consisted of 184 families in the intervention group who attended the Chancenreich program, and 58 families in the comparison group who did not participate in the program. At T1 the children had an average age of 41 months old. The same families were asked to participate in the study again 24 months later (T2). Children were now an average of 68 months old. For T2 66% (*N* = 121) of the families from T1 also participated in the second point of measurement in our study, while 71% (*N* = 41) of the families from the comparison group participated at T2. In order to check whether the composition of the sample has changed significantly, the families dropped out of the study were examined with regard to certain characteristics such as poverty, migration background and the mother’s university degree. Significant differences were found between the families that participated at the second measurement point and those that no longer participated. In the group of families who didn’t participate at the study anymore the proportion of poor families was twice as high (33%) as in the group of families that remained in the study. The change in the sample composition led to the tendency for the comparison group and the Chancenreich group to converge in their socio-structural composition. The overall response rate of 68.5% can be considered very good for such studies. Table 1 shows the descriptive statistics for child and family characteristics by both groups at the second point of measurement. The specific composition of the intervention and comparison group must be taken into account when interpreting the results.

**TABLE 1:** Descriptive statistics for familial and individual children’s characteristics by intervention and comparison group for the second point of measurement.

	Intervention group	Comparison group		
	%/*M (SD)*	%/*M (SD)*	*t-*tests	χ^2^- tests
Age	68.98 (5.55)	63.51 (8.36)	*t*_(157)_ = −4.69***	–
Female	45.45%	53.85%	–	χ^2^_(1)_ = 0.83
First-born	60.50%	69.23%	–	χ^2^_(1)_ = 0.96
Mother graduated from university^a^	36.67%	61.54%	–	χ^2^_(1)_ = 7.44**
Main spoken language at home^b^	22.31%	7.69%	–	χ^2^_(1)_ = 4.14*
Net equivalent household disposable income^c^	1545.87 (551.68)	1656.22 (439.23)	*t*_(__149)_ = 1.12	–
Poverty (<€1,033)	20.35%	7.89%	–	χ^2^_(1)_ = 3.10
Home learning environment	4.15 (0.69)	4.29 (0.50)	–	

n varies between 113 and 121 for the intervention group and between 38 and 39 for the comparison group. ^*a*^1 = yes. ^*b*^1 = not German.^*c*^equivalent household disposable income (Eurostat, 2018). *p < 0.05, **p < 0.01, ***p < 0.001.

### Measures

#### Language Development

Two main indicators that reflect the language development of children were measured by using standardized instruments: the Peabody Picture Vocabulary Test – Third Edition (PPVT-III) and the TROG-D. The German research version of the PPVT (Dunn and Dunn, 1997; Roßbach et al., 2005) captures the child’s receptive vocabulary and was assessed at both points of measurement, that is the ages of 3 and 5. In this test, children were asked to select the correct picture from a set of four pictures for each given word. The test covered 40 items. The PPVT is one of the most widely used instruments of its kind and reports high internal consistency. A mean score of the PPVT was calculated for the analysis with a theoretical range between 0 and 1. In our sample we found ceiling effects at the second point of measurement (Chancenreich group T1: *M* = 0.78, *SD* = 0.21; T2: *M* = 0.96, *SD* = 0.09; Comparison group T1: *M* = 0.81, *SD* = 0.18; T2: *M* = 0.98, *SD* = 0.04). The German version of the TROG (TROG-D; Fox, 2013) assesses the child’s receptive understanding of grammar and was only conducted at the second point of measurement (Chancenreich group: *M* = 60.84, *SD* = 10.43 Comparison group: *M* = 58.68, *SD* = 16.48). This grammar comprehension test examines the understanding of the grammatical structures of the German language, which are marked by inflection, functional words, and sentence order. This is assessed using target sentences and four pictures, one of which matches the target sentence. The German version of the test consists of 21 item groups of four items each (Lüke et al., 2016).

#### Course Formats

As described earlier, the parent-child-interaction focused courses and parenting-skills focused courses are part of the Chancenreich program (and thus relevant for the intervention group) for children up to 3 years old. For that reason, we included the grouping variable in our models of analysis. Further, since these courses do exist on the open market, it cannot be entirely ruled out that comparison group families may attend these courses voluntarily. For that reason, participation in parent-child-interaction focused courses and parenting-skills focused courses was assessed for both the intervention and the comparison group families at the two points of measurement. At the second time of measurement, the Chancenreich families have already completed the program, but they still often attend other courses, as did the families from the comparison group. Additionally, at the second time of measurement, the families were asked about their attendance of early education-focused courses. Parents were asked which courses they had attended exclusively as parents or together with their children, using an open response format. The courses were then summed up and included in the analysis as the number of attended courses. For further analysis, we also calculated the total number of course attendances at the second point of measurement.

Table 2 shows those numbers of courses attended per category by both groups. Both groups attended more parent-child-interaction focused courses when their children were aged 3 (T1) than when they were aged 5 (T2). At the second point of measurement children mostly participated at early-education focused courses. There were no significant differences between the intervention group and the comparison group across all course types.

**TABLE 2:** Descriptive statistics for families’ course participation by intervention and comparison group for the first and second point of measurement.

	Course types	Intervention group^a^	Comparison group^b^	
		*M (SD)*	*M (SD)*	*t-*Test
T1	Parent-child-interaction	0.78 (0.49)	1.03 (0.90)	*t*_(157)_ = 0.03
	Parenting-skills	0.28 (0.49)	0.21 (0.41)	*t*_(157)_ = −0.09
T2	Parent-child-interaction	0.14 (0.35)	0.18 (0.39)	*t*_(158)_ = 0.059
	Parenting-skills	0.12 (0.35)	0.21 (0.41)	*t*_(158)_ = 1.34
	Early-education focused	1.88 (1.51)	2.03 (1.60)	*t*_(158)_ = 0.50

*The *t*-test showed no significant differences by group. ^*a*^n varies between 120 and 121 for the intervention group. ^*b*^*n* = 39.*

#### Control Variables

In order to take into account the differences between the intervention and comparison group and to avoid any confusion of background characteristics with the model predictors, the following general sociodemographic and specific child characteristics were included in all analyses: children’s age and sex, main spoken language at home, net equivalent household disposable income adjusted by the modified equivalent scale of the Organisation for Economic Co-operation and Development (OECD) (Eurostat, 2018), the mother’s highest educational degree, the child’s birth order status, and the family’s HLE. For the latter one, parents were asked how often they engage in home learning activities with their children, ranging between *never* (0) and *every day* (7). The measure consists of 31 items (Cronbach’s α = 0.86) representing the domains numeracy, science, reading, conversation, and creative and practical activities (e.g., ‘Practicing singular numbers or counting together with the child, e.g., counting fingers or throwing dice’).

### Data Analyses

The data was checked for missing data and outliers and missing data patterns were analyzed. The percentage of missing data from the variables was 1.2–5.6%. The MCAR test according to Little (1988) indicated that the missing data was missing completely at random (MCAR; χ^2^ = 43.82, *df* = 31, *p* > 0.05). Under the condition that data is completely missing at random, Full Information Maximum-Likelihood (FIML) approach estimates are the most reasonable method to estimate missing data (Enders and Bandalos, 2001). Even though the amount of missing data was generally low, we conducted the FIML approach in all regression models to minimize bias in parameter estimates (Eid et al., 2013).

With multiple regression analyses, Mahalanobis distance scores were generated. Since two cases were above the Mahalanobis distance threshold of χ^2^(14) = 36.12, they were removed for the following analyses (Tabachnick and Fidell, 2007).

OLS multiple regression analysis were conducted to predict: (1) the attendance rates of different course types at two points of measurements, (2) the attendance rates predicted by family background characteristics, (3) the children’s level of development at the second time of measurement, and to predict (4) the children’s development between the first and second time of measurement. For the latter, the children’s outcomes at the first time of measurement were included in the OLS multiple linear regression models.

For the third and the fourth aims, three OLS regression analyses were conducted for each child outcome: model A includes the grouping variable, where the comparison group served as the reference group; model B includes the families’ total number of attended courses for each of the categories category “parent-child-interaction focused courses” and “parenting-skills focused courses” at the first and second time of measurement. Finally, model C combines models A and B by including the grouping variable, the families’ participation in courses at the first and second time of measurement. For all regression models, the control variables were included in the analysis.

MLR estimator was used because of its robustness according to a violation of the normality assumption (Christ and Schlüter, 2012). All analyses were conducted with Mplus (Version 7.0, Muthén and Muthén, 1998–2012), and SPSS (Version 25.0, IBM Corp, 2017).

## Results

### Course Participation

We first examined the relationship between the number of courses attended and sociodemographic and child characteristics at both times of measurement for the Chancenreich families (Table 3) and the comparison group families (Table 4). The findings presented in Table 3 reveal that children being first-borns (β = 0.23; *SE* = 0.09; *p* = 0.009) and the level of joint activities at home (β = −0.22; *SE* = 0.10; *p* = 0.03) were significant predictors for the number of parent-child-interaction focused courses a Chancenreich family attends at the first time of measurement. Parents are more likely to attend these courses with their first-born children. In addition, parents who report engaging in few joint activities with their children at home also attend these courses more often. At the same time, we find that first born status had a significantly negative regression weight on attendance of parenting-skills focused courses (β = −0.32; *SE* = 0.09; *p* = 0.001). After the Chancenreich program has ended, these families still attend courses regardless of their educational background and income. However, we find income to be a significant positive predictor for the attendance of early-educational courses (β = 0.24; *SE* = 0.07; *p* = 0.005). Furthermore, children from families whose predominant language at home is not German attend fewer parent-child-interaction focused courses after completing the program (β = −0.14; *SE* = 0.07; *p* = 0.04).

**TABLE 3:** OLS regression analysis of number of courses attended by the parents of the Chancenreich program according to sociodemographic and children’s characteristics.

	T1		T2		
	Parent-child-	Parenting-skills	Parent-child-	Parenting-skills	Early-educational
	interaction courses	courses	interaction courses	courses	courses
		
Characteristics	β	β	β	β	β
Child’s age	−0.16(0.10)	0.13 (0.08)	−0.13(0.08)	−0.06(0.10)	0.24**(0.07)
Female	0.06 (0.09)	−0.07(0.09)	0.04 (0.09)	−0.13(0.07)	0.18*(0.08)
First-born	0.23**(0.09)	−0.32**(0.09)	0.13 (0.08)	−0.03(0.10)	0.07 (0.08)
Mother graduated from university^a^	0.11 (0.09)	−0.05(0.09)	0.16 (0.10)	−0.09(0.78)	0.09 (0.09)
Net equivalent household disposable income^b^	0.06 (0.11)	0.00 (0.10)	0.02 (0.10)	−0.12(0.10)	0.24**(0.09)
Main spoken language at home^c^	0.09 (0.11)	−0.10(0.09)	−0.14*(0.07)	−0.07(0.11)	−0.08(0.08)
HLE	−0.22*(0.10)	0.14 (0.08)	0.04 (0.09)	0.24*(0.10)	0.07 (0.07)
*R*^2^ (SE)	0.13*(0.06)	0.13*(0.05)	0.10*(0.04)	0.10 (0.06)	0.24***(0.07)

Standard errors are in parentheses. ^*a*^1 = yes; ^b^equivalent household disposable income (Eurostat, 2018); ^*c*^1 = not German. *n* = 121. *p < 0.05; **p < 0.01; ***p < 0.001.

**TABLE 4:** OLS Regression analysis of number of courses attended by the parents of the comparison group according to child and sociodemographic characteristics.

	T1		T2		
	Parent-child-	Parenting-skills	Parent-child-	Parenting-skills	Early-educational
	interaction courses	courses	interaction courses	courses	courses
		
Characteristics	β	β	β	β	β
Child’s age	−0.23(0.12)	0.18 (0.12)	0.03 (0.13)	−0.01(0.14)	0.26 (0.15)
Female	0.34**(0.11)	−0.19(0.17)	0.09 (0.19)	0.08 (0.14)	0.12 (0.17)
First-born	0.47***(0.13)	−0.01(0.16)	0.18 (0.15)	−0.08(0.17)	0.40**(0.13)
Mother graduated from university^a^	0.36**(0.11)	−0.11(0.15)	0.29 (0.17)	0.26*(0.13)	0.12 (0.18)
Net equivalent household disposable income^b^	0.23 (0.12)	0.24 (0.14)	−0.05(0.14)	0.23 (0.15)	0.02 (0.16)
Main spoken language at home^c^	−0.02(0.15)	0.30 (0.16)	−0.20*(0.08)	0.03 (0.19)	0.07 (0.10)
HLE	0.13 (0.13)	0.12 (0.16)	0.32**(0.11)	0.13 (0.11)	0.10 (0.14)
*R*^2^ (SE)	0.52***(0.11)	0.24 (0.12)	0.17 (0.11)	0.13 (0.11)	0.19 (0.11)

Standard errors are in parentheses. ^*a*^1 = yes. ^*b*^ equivalent household disposable income (Eurostat, 2018). ^*c*^1 = not German. *n* = 39. *p < 0.05; **p < 0.01; ***p < 0.001.

Among the families in the comparison group (Table 4), we see a slightly different pattern compared to the Chancenreich families. When predicting the number of attended parent-child-interaction courses at the first point of measurement the mother’s highest educational level was a significant predictor (β = 0.36; *SE* = 0.11; *p* = 0.001). Furthermore, mothers with a higher educational level attend parenting-skills courses more frequently at the second time of measurement when the children are aged 5 (β = 0.26; *SE* = 0.13; *p* = 0.04). Similar to the Chancenreich families, we found that parents of 5-year-old children from the comparison group attend few parent-child-interaction focused courses if the main language spoken at home is not German (β = −0.20; *SE* = 0.08; *p* = 0.02) and if they engage in a higher number of joint activities with their child at home (β = 0.32; *SE* = 0.11; *p* = 0.003).

This means that we do indeed find different patterns in attendance rates with regard to child and family characteristics. This illustrates that socio-economic aspects (e.g., the mother’s education) for families in the comparison group are predictive of participation in such courses. For the Chancenreich families the quality of HLE is more relevant.

Regarding the second research question, we asked what relationships exist between the attendance of the Chancenreich program and further attendance of courses after completion of the program. We conducted five OLS multiple regression models for the number of attended courses at T2 on course attendance at T1, presented as rows in Table 5. The first model (M1) includes as a predictor the parent-child-interaction focused courses at the first point of measurement; M2 includes the parenting-skills focused courses at T1; M3 includes only the group variable; M4 includes the parent-child-interaction focused courses and group variables; and M5 includes the parenting-skills focused courses and the group variable. We controlled for family and child characteristics in all conducted regression models.

**TABLE 5:** OLS Multiple Regression analysis of number of attended courses by the parents at T2 on participation in Chancenreich program and courses participation at T1.

	Number of courses T2								
		Parent-child-		Parenting-skills		Early-education		Total number	
		interaction-courses		courses		courses		of courses	
T1		β	*R*^2^	β	*R*^2^	β	*R*^2^	β	*R*^2^
M1	Parent-child-interaction courses	−0.09(0.08)	0.10**(0.04)	0.14 (0.09)	0.07 (0.04)	0.09 (0.08)	0.20***(0.05)	0.10 (0.08)	0.20***(0.06)
M2	Parenting-skills courses	0.14 (0.09)	0.11**(0.04)	0.02 (0.08)	0.05 (0.04)	0.04 (0.07)	0.19***(0.05)	0.08 (0.07)	0.19***(0.05)
M3	Group (1 = Chancen-reich)	0.07 (0.09)	0.10*(0.04)	−0.08(0.09)	0.06 (0.03)	−0.05(0.09)	0.19***(0.05)	−.05(0.08)	0.19***(0.05)
M4	Parent-child-interaction courses	−0.09(0.07)	0.10**(0.04)	0.14 (0.09)	0.07 (0.04)	0.09 (0.08)	0.20***(0.05)	0.10 (0.08)	0.20***(0.06)
	Group (1 = Chancen-reich)	0.07 (0.09)		−0.08(0.09)		−0.05(0.08)		−0.05(0.08)	
M5	Parenting-skills courses	0.07 (0.09)	0.12**(0.04)	0.02 (0.08)	0.06 (0.04)	0.04 (0.07)	0.19***(0.05)	0.08 (0.07)	0.20***(0.05)
	Group (1 = Chancen-reich)	0.14 (0.09)		−0.08(0.09)		−0.05(0.09)		−0.05(0.08)	

Standard errors are in parentheses. All models control for child’s age, sex, and first-born status, mother’s university degree, equivalent household disposable income, main spoken language at home, and home learning environment. *n* = 160. *p < 0.05; **p < 0.01; ***p < 0.001.

We found no significant association between the attendance of courses at the first and the second time of measurement. This means that the attendance of courses when the children were 3 years old had no effect on the attendance of courses when the children were 5 years old.

### Language-Related Outcomes for Children Aged 5

Following research question three, we examined what effect the Chancenreich program and the different course formats have on children’s levels of language development at the age of 5. Table 6 presents the results of three regression models for each language outcome, both vocabulary (PPVT) and understanding of grammatical structure (TROG-D). No statistically significant differences were found between the children of the Chancenreich group and the comparison group regarding their vocabulary and their understanding of grammatical structure when considering all control variables. However, the attendance of parenting-skills courses is associated with a stronger vocabulary in children at the age of 5 (Model b: β = 0.09; *SE* = 0.03; *p* = 0.005; Model c: β = 0.09; *SE* = 0.03; *p* = 0.009). We found no further relationship regarding the attendance of the other course formats at the first and second point of measurement.

**TABLE 6:** OLS Regression models on the level of vocabulary (PPVT) and understanding of grammar structure (TROG-D).

	PPVT			TROG-D		
	(a)	(b)	(c)	(a)	(b)	(c)
	β	β	β	β	β	β
**Group^a^**	−0.10(0.06)		−0.08(0.06)	0.08 (0.10)		0.07 (0.10)
**Number courses T1**						
Parent-child-interaction courses		−0.02(0.06)	−0.02(0.06)		−0.15(0.13)	−0.15(0.13)
Parenting-skills courses		0.08 (0.05)	0.08 (0.05)		−0.16(0.09)	−0.16(0.09)
**Number courses T2**						
Parent-child-interaction courses		−0.08(0.06)	−0.07(0.06)		0.09 (0.07)	0.08 (0.07)
Parenting-skills courses		0.09**(0.03)	0.09**(0.03)		0.00 (0.06)	0.01 (0.06)
Early-education courses		0.11 (0.06)	0.10 (0.06)		0.06 (0.10)	0.07 (0.10)
***R*^2^ (SE)**	0.13*(0.05)	0.14**(0.05)	0.15**(0.05)	0.08 (0.04)	0.11*(0.06)	0.11*(0.06)

Standard errors are in parentheses. ^*a*^1 = Chancenreich. Models a, b, c: controlling for child’s age, sex, and first-born status, mother’s university degree, equivalent household disposable income, main spoken language at home, and home learning environment. *n* = 160. *p < 0.05; **p < 0.01; ***p < 0.001.

#### Language Development for Children Between the Ages of 3 and 5

Finally, we examined the effects of Chancenreich and the three different course formats on the development of children’s vocabulary skills between the ages of 3 and 5. For this purpose, we added the PPVT score at the first measuring point as a predictor to the previous regression model (see Table 6) in order to interpret the coefficients as effects on development.

The findings in Table 7 show a significant, positive effect of parenting-skills focused courses on the development of vocabulary skills (Model b: β = 0.14; *SE* = 0.06; *p* = 0.02; Model c: β = 0.15; *SE* = 0.06, *p* = 0.01). Children of parents who have attended more parenting-skills focused courses by the age of 3 exhibit better vocabulary development than children whose parents attended fewer courses. No effects were found for the participation in the Chancenreich program and for the attendance of the other course formats at the first and second point of measurement. This means that the effect can only be due to participation in parenting-skills focused courses.

**TABLE 7:** OLS Regression models on vocabulary development (PPVT) between the ages of 3 and 5.

	PPVT		
	(a)	(b)	(c)
	β	β	β
**PPVT T1**	0.58***(0.13)	0.62***(0.11)	0.62***(0.12)
**Group^a^**	0.03 (0.07)		0.05 (0.07)
**Number courses T1**			
Parent-child-interaction courses		−0.07(0.07)	−0.06(0.06)
Parenting-skills courses		0.14*(0.06)	0.15*(0.06)
**Number courses T2**			
Parent-child-interaction courses		−0.08(0.05)	−0.09(0.05)
Parenting-skills courses		0.09*(0.04)	−0.06(0.04)
Early-education courses		0.04 (0.05)	0.05 (0.05)
***R*^2^ (SE)**	0.32**(0.11)	0.37**(0.12)	0.36**(0.11)

Standard errors are in parentheses. ^*a*^1 = Chancenreich. *n* = 160. Models a, b, c: controlling for child’s age, sex, and first-born status, mother’s university degree, equivalent household disposable income, main spoken language at home, and home learning environment. *p < 0.05; **p < 0.01; ***p < 0.001.

## Discussion

Mastering language development is one of the major developmental milestones in early childhood; it plays a key role not only for the ability of children to interact with their social environment, but also impacts their early and later academic success (Hoff, 2006).

According to the theoretical model of the HLE, the structural characteristics of the family and the educational beliefs of the parents are related to process quality, this last element itself being directly related to the child’s outcomes (Kluczniok et al., 2013). Families that are prevented from providing a rich HLE are defined as disadvantaged (Melhuish et al., 2008). Intervention programs are developed to encourage these parents in their theoretical knowledge and in their practical parenting skills. However, there exists little evidence on the long-term effects of family support programs in Germany. For this reason it is interesting to understand how early family support of HLE can affect core language competences (e.g., receptive vocabulary, grammar structure). Chancenreich is one example of a family support program that offers families different services in a modular approach. One of the modules is the parent training module. It consists of courses that focus either on parent-child-interaction or on parenting skills. In this paper we examined, on the one hand, the attendance patterns of families in different course types when the children were 3 and 5 years old, and on the other hand, the effects of the family support program Chancenreich and different course formats – first on children’s vocabulary and understanding of grammatical structure and second on children’s vocabulary development between the ages of 3 and 5, which is considered a core competence of language development in early childhood.

### Course Participation

Many family support programs struggle with hard-to-reach families, e.g., socio-economically deprived families or families with a migration background (Cortis et al., 2009; Boag-Munroe and Evangelou, 2010). For this reason, we first examined the role of both a family’s sociodemographic characteristics and the child’s own characteristics in parental course participation at the first and second point of measurement. The results show a relationship between course participation and several child characteristics. Parents with younger children attend more parent-child-interaction focused courses at the first time of measurement, which can be explained by the content orientation of these courses, which is more appropriate for younger children. In contrast, older children attend early-education focused courses more often. Furthermore, we found a positive ‘first-born effect’ for parent-child-interaction focused courses and parenting-skills focused courses at the first time of measurement, and a positive effect for the number of early-education focused courses attended at the second point of measurement. These results corroborate existing research on parental time investment and the number of siblings: first-born children are, at least for a period in their early lives, by definition the only child in which parents invest their time and resources (Lawson and Mace, 2009). Therefore, parents might have more time to invest in their child’s development and as a result, participate in these courses. Family support programs can address these findings in promoting courses for a second child, or in supporting parents of multiple children by adapting the content of the courses to these particular needs.

Further findings with regard to the Chancenreich families revealed the positive effect of financial resources on the number of early education courses attended at T2, and for the comparison group the positive effect of a higher educational background of the mother on the number of parenting-skills focused courses attended at T1 and T2. The findings confirm existing research on the important role of structural familial characteristics in the use of educational services (Dearing et al., 2009; Carolan, 2018). Both course formats are not developed specially for the Chancenreich program, but are rather offered to all parents on the open educational market of early childhood courses. These courses are well-known and widely used in Germany. While course participation for Chancenreich families is free of charge and do not need to be organized by the parents themselves, the comparison group families would be required to pay for the courses and need to find the courses themselves.

After completing the program, Chancenreich families are still free to choose different course formats on the educational free market. Further, we did not find a significant difference between the Chancenreich and the comparison group regarding the number of the attended courses before and after the program. Since these families are usually hard to reach and persuade to attend courses, these results can be interpreted as a success for the Chancenreich program in the context of the effect of the educational background of the mothers. Against the backdrop of the groups’ differing compositions with regard to socio-economic characteristics (a higher number of disadvantaged families in the Chancenreich program), and confirming our theoretical assumptions, there seems to be a transition effect in terms of early positive experiences with the informal educational system during the Chancenreich program. It might motivate and encourage Chancenreich parents to be further involved in their children’s development, and transferring this motivation to other educational services even after completion of the program. However, Chancenreich families with lower incomes are less likely to attend early-educational courses at T2. It is reasonable to assume that the continued financial support of families in family support programs might encourage parents to let their children participate in this type of course as well.

### Course Participation and Children’s Language Skills

No effects were found for the understanding of grammar at the age of 5, either as an effect of participation (or not) in the Chancenreich program, or for the number of different types of course parents and children attended. However, the number of parenting-skills focused courses parents attend by the time their child is three has a significant, positive effect on the child’s level of vocabulary skills at the age of 5 and on the development of vocabulary skills between the ages of 3 and 5. Specifically, in the light of the positive relationship between the number of parent-child-interaction focused courses attended and the children’s vocabulary levels at age 3 (Wilke et al., 2017), this effect can be interpreted as a sleeper effect. This means that the effects of early participation in parenting-skills focused courses on children’s development remain silent, but were triggered by environmental changes or developmental processes during childhood. We assume that by participating in both course formats, the parent–child interaction is promoted in different ways. Courses that parents attend together with their children directly stimulate interaction and communication. Courses that focus on parenting skills indirectly encourage parents to become more involved with their children and to establish or expand a more positive and beneficial communication. Furthermore, these effects might reflect motivational or attitudinal changes, changes in perception of parental self-efficacy, or the reduction of barriers to effective positive parenting, all of which have long-term, ongoing effects on children’s outcomes (Sandler et al., 2011). The overall findings show that motivating parents to participate in a family support program is only one side of the coin; the other is the content orientation of the program and the actual activities of the parents during the program, which have a significant impact on child language development.

### Limitations

The results must be interpreted with regard to the restriction of the study design, the sample size, the selection bias of the groups, and the applied measures. The study is designed as a quasi-experimental study with an intervention group and a comparison group. A randomization of the groups could not be carried out due to ethical considerations and recruitment strategies. This led to a lower control of possible side conditions and to problems with sample bias. In comparison, a quasi-experimental design has a greater external validity, and it gave us the opportunity to achieve greater accessibility for the participants.

We countered the decreasing sample size, due to random drop out and missings, by conducting FIML approaches in all models.

Furthermore, the standardized PPVT (Dunn and Dunn, 1997; Roßbach et al., 2005) was used to assess the children’s vocabulary of the children at both points of measurement – ages 3 and 5. We apply the same items at both measurement points, which has certain theoretical advantages, but also means the PPTV shows ceiling effects for the older age group; this results in less variance for this measure, as well as a reduction in the ability to differentiate at the upper level of the vocabulary competences.

Another limitation is the group composition. The comparison group contains families with, on average, mothers with higher educational levels. At the second point of measurement, the Chancenreich group lost migrant families and families with lower incomes. Hence, the groups converge in comparison to the first point of measurement. Nevertheless, this limitation must be taken into account when interpreting the results.

### Implications

This study is one the few studies worldwide and the first study in Germany to examine the long-term impact of family support programs and the different types of courses on offer. We found positive effects over time of parents’ attendance of parenting-skills focused courses on their children’s level and development of vocabulary skills. In the context of the theoretical model of the HLE, this indicates that these courses might improve both parental beliefs and process quality, thus positively influencing the development of their children. However, further research should focus on that mechanism and the processes of choosing different course types and the effectiveness of the quality of the courses (quantity of parental attendance and quality of the content of the courses). Additionally, research should examine a broader range of outcomes, including children’s social and emotional well-being.

Finally, we found no direct effect of participation in courses of younger children on a later higher rate of course attendance rate in children of preschool age. Further research is needed to investigate if and how early parental contact with the informal educational system affects their educational aspirations, and perhaps reduces barriers to later parental involvement in their children’s development in both formal and informal contexts.

With regard to practical implications, monitoring is particularly needed with regard to the content and high-quality implementation of such courses. Programs are particularly successful if they manage to continuously develop content according to the needs and expectations of the target families. In order to ensure a high quality implementation of programme content, the role of professional competencies must be taken into account. In addition, Chancenreich as a local program is a typical example for the system of family support services in Germany. At the same time, however, it is also a role model for other programs when it comes to reviewing and developing programme content through summative and formative evaluations.

## Data Availability Statement

The dataset generated for this study will not be made publicly available. Due to the sensitive nature of the questions asked in this study, study participants were assured data would remain confidential and would not be shared. Requests to access the data should be directed to corresponding author, FC.

## Ethics Statement

Ethical approval was not required for this study. The study was consistent with the ethical principles of human subjects. The parents were informed about the confidentiality and anonymity of their data. Participating parents signed the informed consent on a voluntary basis.

## Author Contributions

All persons who meet authorship criteria are listed as authors, and all authors certify that they have participated sufficiently in the work. FC made substantial contributions to the data analysis and interpretation, and to the conception and design of the article, drafting the article and revising it critically. JS made substantial contributions to data analysis and interpretation, and to drafting and revising the article. EV collected the data and contributed to the data analysis. YA made substantial contributions to the conception and design of the study and critically revised and approved the final version to be published.

## Conflict of Interest

The authors declare that the research was conducted in the absence of any commercial or financial relationships that could be construed as a potential conflict of interest.

## Footnotes

1 In the year of the first measurement point of the surveys, 2014, the ECEC rates for the federal state North Rhine-Westphalia were 23.8% for children under the age of two and 92.4% for children over the age of three (https://www.destatis.de/DE/ZahlenFakten/GesellschaftStaat/Soziales/Sozialleistungen/Kindertagesbetreuung/Tabellen/Tabellen_Betreuungsquote.html;jsessionid=32D1E08EC2C8E317BD27EC8B20C1F934.InternetLive1, 07.12.2018).

2 Usually one caregiver participates in parent-child-interaction courses, typically the mother.

3 The comparison group was recruited in another town because almost all families in the town where Chancenreich is implemented took part in the program.
